# Tracking Bone Loss in GLP-1RA Therapy: The Potential of the Deoxypyridinoline Urine Test

**DOI:** 10.3390/diagnostics16132128

**Published:** 2026-07-07

**Authors:** Angeliki Margoni, Efthimia K. Basdra, Athanasios G. Papavassiliou

**Affiliations:** Department of Biological Chemistry, Medical School, National and Kapodistrian University of Athens, 11527 Athens, Greece; mmargoni@med.uoa.gr (A.M.); ebasdra@med.uoa.gr (E.K.B.)

**Keywords:** glucagon-like peptide-1 receptor agonists (GLP-1RAs), bone turnover, deoxypyridinoline (DPD), dynamic biomarkers, osteoporosis, precision monitoring, prognostic stratification

## Abstract

Skeletal safety of glucagon-like peptide-1 receptor agonists (GLP-1RAs) remains uncharted, with emerging evidence suggesting a divergence between mono- and dual-agonist therapies. GLP-1RA monotherapy appears bone-neutral, with modest or no adverse effects on bone mineral density (BMD), whilst dual agonists may confer a relatively higher risk of osteoporosis and fractures, plausibly mediated by greater weight loss magnitude and concomitant reductions in lean body mass (LBM) rather than direct osteotoxicity. Intensified surveillance is warranted in susceptible phenotypes, including older adults and postmenopausal women with low baseline BMD under conditions of rapid weight loss. Osteoporosis risk is further amplified by pre-existing osteopenia, nutritional deficiencies, and concomitant exposure to bone-active agents. Given the limitations of serial dual-energy X-ray absorptiometry (DXA), including cumulative radiation exposure and limited sensitivity to early remodeling changes, biochemical markers potentially depict bone turnover more dynamically. Measurement of dynamic bone resorption markers enables early identification of skeletal disturbances, supporting proactive adjustment of therapeutic strategy, dosing, and duration. Specifically, deoxypyridinoline (DPD), a bone-specific collagen crosslink, is a highly sensitive and rapidly responsive urine biomarker of osteoclastic activity. Incorporating DPD urine testing into monitoring frameworks potentially facilitates individualized therapeutic modulation, optimizing the metabolic efficacy of GLP-1RAs while safeguarding skeletal integrity.

## 1. Introduction

The global rise of obesity has become one of the defining public health crises of recent decades, paralleled by a dramatic escalation in type 2 diabetes mellitus (T2DM). Driven by excess adiposity, sedentary behavior, and progressive metabolic dysregulation, these conditions converge into the phenotype of “diabesity”, in which insulin resistance, adipose tissue dysfunction, chronic low-grade inflammation, and cardiometabolic risk are mutually reinforced [1]. Historically, higher body weight was considered protective for bone through increased mechanical loading; however, this paradigm has been challenged by evidence linking obesity—particularly visceral and ectopic adiposity—to impaired bone quality, altered bone turnover, osteoporosis, and increased susceptibility to fragility fractures [1,2,3]. The skeletal consequences of obesity are multifactorial and reflect the interaction between biomechanical forces, adipose tissue distribution, nutritional status, inflammatory mediators, adipokines, sex steroids, and hormones involved in bone metabolism [1,2,3]. In parallel, T2DM further compounds skeletal vulnerability through mechanisms that are not fully captured by conventional bone mineral density (BMD) assessment. Patients with T2DM often exhibit preserved or even increased BMD yet paradoxically experience an elevated fracture risk [4,5,6,7,8]. This dissociation reflects diabetes-related deterioration in bone quality, driven by chronic hyperglycemia, accumulation of advanced glycation end-products (AGEs) within the bone matrix, oxidative stress, microvascular dysfunction, and persistent low-grade inflammation [4,5,6,7,8]. These processes impair osteoblast function, collagen integrity, bone material properties, and skeletal microarchitecture, collectively contributing to reduced bone strength despite apparently reassuring densitometric findings [9,10].

Recent advances in pharmaceutical engineering have transformed the therapeutic landscape of diabesity by incretin-based therapies, particularly glucagon-like peptide-1 receptor agonists (GLP-1RAs) and newer dual glucose-dependent insulinotropic polypeptide (GIP)/GLP-1 receptor agonists [11,12,13,14]. These agents target key mechanisms underlying insulin resistance, appetite dysregulation, and excess adiposity, producing clinically meaningful improvements in glycemic control, weight reduction, and cardiovascular risk [11,12,13,14]. The expanding use of GLP-1RAs and next-generation incretin therapies beyond traditional diabetes indications has created a new clinical imperative: to clarify long-term adverse effects on the skeleton that remain insufficiently elucidated [13,14,15]. Emerging evidence suggests that skeletal effects may vary by pharmacologic class: GLP-1RA monotherapy appears largely bone-neutral, whereas dual agonists may be associated with increased osteoporosis and fracture risk [11,12,13,14,15,16]. This divergence likely reflects competing biological forces: treatment-induced weight loss may reduce mechanical loading and accelerate bone loss, while improved glycemic control, reduced inflammation, and direct GLP-1RA-mediated actions on bone cells may exert neutral or potentially protective skeletal effects [11,12,13,14,15]. Herein, we advocate for a clear distinction between skeletal alterations attributable to obesity and T2DM and those emerging as direct or indirect consequences of pharmacological intervention. Systematic tracking of bone health in individuals receiving GLP-1RAs is essential to determine whether these agents preserve, compromise, or modify skeletal integrity during sustained metabolic improvement and weight reduction. Ultimately, because fragility fractures represent the clinically meaningful endpoint of skeletal deterioration, future surveillance strategies should aim not only to detect changes in BMD and bone turnover markers but also to determine whether these alterations translate into differences in fracture incidence. Herein, defining the independent and synergistic contributions of diabesity, weight loss, and incretin pharmacotherapy to skeletal health will be critical for refining patient selection and advancing precision prescribing of GLP-1RAs and next-generation anti-obesity and antidiabetic agents.

## 2. The Skeletal Paradox of GLP-1 Therapies: Protection or Fragility?

Preclinical evidence indicates that incretin-based therapies exert biological and toxicological heterogeneity, thereby differentiating GLP-1R monoagonists from dual agonists regarding skeletal safety [16]. GLP-1R activation—exemplified by liraglutide and semaglutide—may exert osteoprotective or bone-neutral effects through modulation of key bone remodeling pathways and coordinated regulation of osteoblast, osteoclast, and osteocyte function [11,12,17]. Proposed mechanisms include promotion of osteoblast differentiation, attenuation of osteoclastogenesis, upregulation of osteoprotegerin-mediated inhibition of receptor activator of nuclear factor κB ligand (RANKL) signaling, interaction with the Wnt/β-catenin pathway, suppression of inflammatory mediators, and favorable shifts in bone turnover markers (BTMs), including reductions in C-terminal telopeptide of type I collagen (CTX) alongside increases in procollagen type 1 N-terminal propeptide (P1NP) [9,10,17,18,19]. These findings are supported by experimental models of both diabetes and glucocorticoid-induced osteoporosis, in which GLP-1R activation has been associated with improvements in BMD, trabecular microarchitecture, and skeletal strength [16]. Nevertheless, translation into human physiology remains inconsistent. Randomized clinical trials generally demonstrate neutral skeletal outcomes, although some evidence suggests enhanced bone formation responses during weight reduction, implying that GLP-1RAs may partially counterbalance catabolic remodeling under conditions of energy deficit and metabolic stress [11,12,13,14,15,17,18,19].

In contrast, dual agonists targeting GLP-1/GIP receptors appear to induce a more complex and potentially unfavorable skeletal phenotype [13,18,19]. Accumulating large-scale data suggest a comparatively higher risk of osteoporosis and fragility fractures associated with agents such as tirzepatide [20,21]. The strongest direct evidence derives from a TriNetX cohort of approximately 460,000 individuals, in which tirzepatide exposure was associated with higher risks of osteoporosis and fractures than GLP-1RA monotherapy or non-use, with hazard ratios (HRs) of 1.44 and 1.48, respectively [22]. Importantly, these effects are unlikely to reflect direct osteotoxicity, but rather the skeletal consequences of rapid, substantial weight loss, accompanied by disproportionate reductions in lean body mass (LBM) and mechanical loading that disrupt bone turnover balance, distinguishing them from the generally bone-neutral profile of GLP-1R monoagonists [11,12,13,18,20,21,22,23].

Mechanistically, this may promote a high-resorption bone phenotype through altered endocrine signaling, nutritional status, muscle–bone crosstalk, adipokine activity, and gut–bone axis regulation [11,12,13,20,21,22]. Similarly, comparative BMD data for semaglutide versus tirzepatide further support weight-loss magnitude as the principal driver of skeletal deterioration, with more pronounced effects in non-diabetic individuals receiving potent GLP-1RA-based therapies [23]. This reinforces the view that skeletal vulnerability depicts accelerated catabolic adaptation rather than receptor-specific osteotoxicity, although the greater metabolic potency of dual agonism may indirectly amplify fragility beyond skeletal compensatory capacity [18,19,20,21,22,23]. Future triple agonists could potentially augment this trajectory further, as escalating metabolic efficacy may outpace skeletal adaptation and repair mechanisms. Beyond distinct pharmacodynamic profiles, incretin-based therapies may differentially affect downstream skeletal pathways, including osteoblast–osteoclast coupling, adipose–bone crosstalk, and muscle preservation. Accordingly, these agents represent a heterogeneous class with divergent skeletal effects, warranting stratified osteoporosis risk assessment during rapid weight loss, particularly in susceptible individuals.

## 3. Redefining Skeletal Vulnerability in the Era of GLP-1R Agonism

High-risk populations for osteoporosis and fragility fractures are no longer confined to postmenopausal women with low BMD, but increasingly encompass a broad spectrum of metabolically, therapeutically, and systemically vulnerable individuals [24]. Contemporary recommendations from the National Osteoporosis Guideline Group (NOGG) and other international societies recognize osteoporosis as a complex multisystem disorder arising from the interplay between ageing, chronic inflammation, endocrine and metabolic dysregulation, malignancy, nutritional imbalance, sarcopenia, and prolonged pharmacologic exposure [23,24,25,26]. Consequently, fracture risk stratification has progressively shifted beyond isolated densitometric thresholds toward integrated clinical risk assessment models, particularly in individuals exhibiting elevated fracture risk assessment (FRAX^®^) tool or QFracture scores despite preserved or apparently normal serial dual-energy X-ray absorptiometry (DXA)-derived BMD values [23,24,25,26]. Noteworthy, mounting evidence indicates that diabetic bone disease is characterized by qualitative skeletal impairment, including cortical porosity, trabecular microarchitectural disruption, altered collagen cross-linking, suppressed bone turnover, and compromised biomechanical competence. These abnormalities provide a plausible explanation for the paradoxical increase in fracture risk observed despite non-osteoporotic BMD values and underscore the need for upgraded skeletal surveillance in T2DM [4,5,6,7,8]. Importantly, prolonged disease duration, insulin dependence, chronic low-grade inflammation, sarcopenia, and coexisting obesity further amplify skeletal susceptibility, thereby reducing the diagnostic sensitivity and clinical utility of conventional densitometric assessment in these populations [23,24,25]. Furthermore, reduced caloric intake accompanying GLP-1RA-induced weight loss may predispose susceptible individuals to inadequate protein, calcium, and vitamin D intake, thereby compromising muscle preservation, impairing bone remodeling, and potentially amplifying skeletal fragility, particularly in older adults and patients with pre-existing nutritional deficiencies [23,24,25].

Equally important is the expanding burden of medication-triggered osteoporosis, now recognized as one of the leading causes of secondary skeletal deterioration worldwide [26]. High-risk pharmacologic categories include glucocorticoids, endocrine therapies, anticonvulsants, anticoagulants, anticancer agents, antidiabetic therapies, thyroid hormone replacement, proton pump inhibitors, antiretroviral agents, antidepressants, and diuretics [26]. Long-term glucocorticoid exposure remains among the most devastating secondary causes of osteoporosis owing to profound suppression of osteoblastogenesis, enhancement of osteoclast-mediated bone resorption, impairment of osteocyte viability, and rapid deterioration of trabecular microarchitecture, ultimately resulting in substantial fracture-related morbidity [27,28]. Additionally, the growing trend of incretin-based therapies, particularly dual agonists such as tirzepatide, require careful skeletal vigilance as recent cohort analyses have reported a modest but measurable increase in fracture risk among GLP-1RA users compared to alternative antidiabetic drugs, including sodium–glucose cotransporter-2 (SGLT2) inhibitors and dipeptidyl peptidase-4 (DPP-4; also known as CD26) inhibitors [11,12,13,14,15,16,17,18,19,20,21,22]. Similarly, patients undergoing oncologic therapies represent another critically vulnerable population. Endocrine treatment, chemotherapy, radiotherapy, and human epidermal growth factor receptor (HER)-family targeted therapies have all been associated with accelerated skeletal decline and increased fracture burden [26]. Notably, HER3 signaling has emerged as a potential mechanistic contributor not only to HER3-positive breast cancer progression and bone metastasis, but also to cancer-associated bone loss through disruption of bone remodeling homeostasis and amplification of osteolytic pathways that frequently lead to a devastating form of osteoporosis [29,30]. Considering that a 5-year study presented at the American Academy of Orthopaedic Surgeons (AAOS) showed that GLP-1RA users had a 29% higher risk of osteoporosis compared to non-users, patients undergoing anti-HER3 therapies and concurrently taking GLP-1RAs should be under intensified surveillance [30]. Accordingly, skeletal surveillance should be individualized according to baseline fracture risk, with intensified monitoring considered for older adults, postmenopausal women, individuals with established osteoporosis or chronic kidney disease, and other patients at increased risk of accelerated bone loss during incretin-based therapy [30].

Against this background, the identification and prioritization of individuals at the highest skeletal risk is of utmost clinical importance. Establishing targeted surveillance strategies focused on carefully selected high-risk populations is equally essential for improving diagnostic precision, enabling timely and effective therapeutic intervention, and ensuring economically sustainable monitoring frameworks. In an era characterized by escalating prevalence of chronic metabolic disease, malignancies, ageing populations, and expanding exposure to long-term pharmacologic therapies, precision-based and risk-adapted osteoporosis surveillance may represent the most clinically effective and cost-efficient approach for reducing fracture burden and associated healthcare expenditures.

## 4. Beyond DXA: Addressing the Skeletal Complexity of Incretin Therapies Through Multidimensional-Tailored Monitoring

Understanding the differential skeletal effects of individual incretin-based therapies has become increasingly important for the development of precision-oriented, clinically meaningful, and economically sustainable monitoring strategies. DXA remains the gold-standard imaging modality for the diagnosis, longitudinal monitoring, and therapeutic assessment of osteoporosis, owing to its widespread availability, reproducibility, relatively low radiation exposure, and robust association between BMD and fracture risk (Table 1) [31,32]. However, the updated 2025 DXA Practice Guideline emphasizes that the clinical utility of DXA is highly dependent on rigorous standardization of acquisition, analysis, and interpretation procedures [31,32]. Multiple technical limitations may significantly affect measurement accuracy and reproducibility, including patient positioning, instrument calibration, motion artifacts, degenerative skeletal changes, vascular calcifications, obesity-related soft tissue interference, and inter-device variability [31,32]. Furthermore, interpretation of DXA findings may be confounded by inconsistencies in reference population selection for T-score and Z-score calculations, inadequate appreciation of least significant change (LSC), and failure to integrate clinical risk modifiers beyond BMD values alone [32]. Although DXA involves minimal ionizing radiation exposure compared with other radiological modalities, cumulative exposure from repeated serial assessments and adjunct imaging procedures should still be considered, particularly in younger patients requiring prolonged monitoring [32]. Given that updated guidelines increasingly acknowledge the limitations of BMD as a standalone surrogate of skeletal fragility, greater emphasis is placed on adjunctive non-BMD modalities. Accordingly, assessment of bone quality should complement conventional densitometry, with trabecular bone score (TBS) providing information on trabecular microarchitecture, vertebral fracture assessment (VFA) detecting occult vertebral fractures, hip structural analysis (HSA) evaluating biomechanical strength, and HR-pQCT offering high-resolution characterization of cortical and trabecular bone architecture beyond BMD alone (Table 1) [10,11,12,31,33]. Additionally, as dual incretin agonists may induce disproportionate LBM loss, skeletal surveillance should extend beyond BMD and BTMs to include assessment of muscle function, sarcopenia, and falls risk, which independently contribute to fragility fracture risk. On a research basis, incorporation of radiological and biochemical phenotyping approaches is required to fathom the underlying mechanisms of osteoporosis and distinguish disease-driven skeletal deterioration from drug-related effects on bone metabolism. Modern multimodal biomarker profiling—including P1NP, CTX, osteocalcin (OC), sclerostin (SOST), parathyroid hormone (PTH), calcium homeostasis parameters, vitamin D, and inflammatory cytokines—offers a far more comprehensive characterization of skeletal remodeling, microarchitectural integrity, endocrine regulation, and inflammation-driven bone deterioration (Table 1) [34,35,36,37].

In parallel, longitudinal assessment of BTMs could facilitate the identification of distinct remodeling signatures associated with obesity, T2DM, and specific incretin-based therapeutic classes. Among these biomarkers, serum P1NP is generally considered the most clinically versatile and preferable for longitudinal osteoporosis management [38,39]. As a direct indicator of type I collagen synthesis and osteoblastic activity, P1NP demonstrates low biological variability, minimal circadian fluctuation, relative stability in renal impairment compared with resorption markers, and excellent responsiveness to anabolic and antiresorptive therapies (Table 1) [37,38,39]. Remarkably, changes in P1NP can be detected within weeks to months after treatment initiation, preceding measurable DXA changes and thereby enabling early assessment of therapeutic efficacy and treatment adherence. Furthermore, international standardization initiatives have markedly improved inter-assay comparability, facilitating its integration into routine clinical practice algorithms [34,35,36,37,38,39].

Additionally, serum β-CTX remains the preferred marker for evaluating osteoclastic activity and rapid changes in bone resorption dynamics [37,38,39]. It is particularly valuable for monitoring antiresorptive treatment response, identifying accelerated bone loss, and assessing rebound remodeling after discontinuation of therapy. Nevertheless, CTX is characterized by substantial circadian and feeding-related variability, necessitating strict fasting morning sampling to ensure analytical reliability. Moreover, renal dysfunction may significantly influence circulating CTX concentrations, limiting interpretability in elderly individuals and patients with chronic comorbidities such as T2DM or chronic kidney disease (CKD) (Table 1) [34,35,36,37,38,39]. Despite these limitations, CTX remains highly sensitive to therapeutic modulation and continues to represent one of the most clinically validated fracture-associated resorption biomarkers [37,38,39]. Among other biomarkers, OC further contributes insights into osteoblast activity and the increasingly recognized interface between skeletal and metabolic regulation, whereas SOST reflects osteocyte-mediated suppression of the Wnt/β-catenin signaling pathway and may serve as a surrogate of impaired mechanotransduction and skeletal aging [34,37,38,39].

Simultaneously, PTH, calcium-phosphate balance, and vitamin D metabolites provide critical information regarding systemic mineral homeostasis and secondary endocrine contributors to skeletal fragility. Importantly, inflammatory cytokines—including interleukin (IL)-6, tumor necrosis factor alpha (TNF-α), RANKL, and other mediators—have gained relevance in chronic inflammatory, metabolic, oncologic, and obesity-related states, where low-grade systemic inflammation profoundly alters bone remodeling kinetics (Table 1) [19,40]. Finally, periostin (POSTN or osteoblast-specific factor 2 (OSF-2)) represents a fundamentally different category of biomarker, reflecting extracellular matrix (ECM) remodeling, osteocyte signaling, and cortical bone integrity rather than classical turnover alone. Recent data suggest that elevated POSTN levels may predict non-vertebral and cortical fragility fractures independently of BMD, particularly in postmenopausal women, showcasing it as a potentially valuable biomarker for identifying alterations in bone quality and microarchitectural deterioration that may not be captured by traditional remodeling markers [41,42]. However, despite its mechanistic attractiveness, POSTN remains largely investigational due to limited assay standardization, insufficient longitudinal validation, heterogeneous study findings, and lack of universally accepted reference ranges. Moreover, POSTN levels may be influenced by cardiovascular, pulmonary, fibrotic, and inflammatory disorders, thereby reducing skeletal specificity [41,42].

## 5. Revisiting Urinary Deoxypyridinoline (DPD): Toward Patient-Friendly Monitoring of Bone Resorption

Urinary DPD, although historically overshadowed by serum biomarkers, reflects a biologically relevant index of bone matrix degradation. This underestimated yet clinically valuable dynamic marker of bone resorption is potentially more patient-friendly for serial assessment due to its urinary origin. As a non-invasive biomarker reflecting collagen degradation and osteoclastic activity, DPD offers practical advantages that may facilitate patient acceptance and adherence to longitudinal monitoring strategies, particularly in outpatient and community-based settings where repeated venipuncture may reduce compliance [43,44,45,46]. Its sensitivity to increased bone turnover may allow detection of metabolic skeletal alterations before overt densitometric decline becomes evident, supporting its clinical utility as an early dynamic indicator of skeletal remodeling (Table 1) [45]. Despite its biological rationale and practical advantages, urinary DPD has not yet been validated as a surrogate endpoint for fracture risk in patients receiving GLP-1 receptor agonists or dual incretin therapies. Future prospective longitudinal studies should determine whether treatment-induced changes in urinary DPD correlate with clinically relevant skeletal outcomes, including incident fragility fractures, vertebral fracture progression, deterioration in TBS, HR-pQCT-derived microarchitectural changes, and longitudinal BMD loss. Such studies should also establish optimal sampling protocols, evaluate intra-individual variability under standardized conditions, define clinically meaningful change thresholds, and compare the predictive performance of urinary DPD with established serum biomarkers including CTX and P1NP. Ultimately, integration of urinary DPD into routine osteoporosis surveillance should depend not only on analytical performance but also on demonstration of incremental prognostic value, cost-effectiveness, and patient acceptability within multimodal fracture-risk prediction strategies. Moreover, several limitations currently restrict the broader clinical implementation of urinary DPD, including its dependence on renal function, hydration status, urinary dilution, physical activity, and circadian variation, all of which contribute to moderate intra-individual biological variability (approximately 20–30% for repeated measurements) and reduce the reliability of isolated determinations [36,37,47]. In contrast to serum CTX and P1NP, urinary DPD also lacks internationally standardized assays, universally accepted reference intervals, and validated clinical cut-off values for fracture prediction or treatment monitoring [47]. These factors may reduce reproducibility and complicate interpretation, particularly in elderly populations, patients with T2DM, and individuals with CKD, in whom renal dysfunction may substantially alter urinary excretion patterns [47]. Consequently, urinary DPD should currently be regarded as a complementary rather than a substitute biomarker, with its greatest potential lying in serial longitudinal assessment under standardized sampling conditions, where changes from an individual’s baseline may provide more meaningful information than absolute values. Although DPD cannot currently replace standardized serum markers such as CTX or P1NP, it may still provide meaningful adjunctive information as a convenient, dynamic, and patient-friendly biomarker in selected clinical contexts [46,47]. Indisputably, the ease and non-invasive nature of urine collection may enhance monitoring feasibility in younger individuals, oncological patients, and postmenopausal women requiring frequent reassessment, or populations reluctant to undergo repeated blood sampling. In an era increasingly focused on patient-centered and accessible, even self-monitoring approaches, the potential role of DPD measurement may therefore deserve renewed consideration within individualized osteoporosis assessment strategies. Future innovation could further expand the clinical relevance of urinary DPD through the development of rapid point-of-care immunochromatographic assays, conceptually analogous to home pregnancy tests [48]. Such low-cost, easy-to-use urine-based immunochemical devices could potentially enable real-time self-monitoring of bone resorption dynamics outside traditional healthcare settings, thereby transforming osteoporosis follow-up into a more accessible and patient-empowered process [48]. This strategy could be conceptually aligned with the established model of home glucose monitoring in diabetes care, whereby repeated patient-performed measurements provide clinically meaningful information beyond isolated clinic-based assessments. In this context, urinary DPD self-monitoring may represent a pragmatic extension of diabetes self-management principles to skeletal health, particularly for patients already familiar with routine biomarker tracking and longitudinal risk surveillance. Its potential value may be especially pronounced in individuals with T2DM or obesity receiving incretin-based therapies, in whom dynamic changes in bone resorption could offer an early warning signal. Although this approach could represent a disruptive and economically feasible direction for mitigating the prevalence of osteoporosis and its complications, substantial analytical validation and standardization are required before clinical implementation. Collectively, current expert recommendations continue to favor serum P1NP and CTX as reference BTMs because of superior assay harmonization and stronger clinical validation [36,37].

## 6. Conclusions

GLP-1RAs and next-generation incretin therapies introduce a critical skeletal paradox into modern diabesity care. Their euglycemic effects may attenuate hyperglycemia-driven bone toxicity, inflammation, oxidative stress, and abnormal collagen glycation; yet their potent weight-loss effects may simultaneously reduce mechanical loading, accelerate bone turnover, compromise lean mass, and expose latent skeletal fragility. Thus, their net impact on bone is unlikely to be uniformly protective or detrimental, but rather the result of opposing metabolic and biomechanical forces.

This duality demands a more sophisticated model of skeletal surveillance. Particularly in high-risk groups—where fracture risk may be underestimated despite preserved or elevated BMD—precision monitoring should extend beyond DXA to ensure that the cardiometabolic triumph of incretin therapy is not accompanied by silent, preventable skeletal harm. Embedding radiological and biochemical skeletal phenotyping into the routine follow-up of patients receiving incretin therapies may enable earlier detection of subclinical skeletal deterioration and timely adjustment of preventive strategies. From a clinical perspective, skeletal surveillance during incretin-based therapy should follow a risk-adapted rather than universal approach. Baseline evaluation should include fracture-risk assessment, nutritional status, and DXA, complemented by BTMs where appropriate, while longitudinal follow-up may incorporate serial biochemical assessment and repeat imaging in patients at high fracture risk, experiencing substantial weight loss, accelerated bone turnover, or clinical deterioration. Alongside pharmacological management, preservation of skeletal health should emphasize adequate protein, calcium and vitamin D intake, weight-bearing and resistance exercise, maintenance of muscle mass, and falls prevention, particularly because disproportionate lean body mass loss during potent incretin therapy may increase sarcopenia and fracture susceptibility independent of BMD.

Future urine DPD self-testing platforms may facilitate a shift toward out-of-clinic monitoring, offering an accessible, patient-centered model analogous to glucose self-monitoring in diabetes. Indisputably, DPD monitoring should currently be considered investigational rather than routine, because available evidence remains limited by heterogeneous study designs, relatively small cohorts, short follow-up, and reliance on surrogate outcomes such as BTMs and BMD rather than incident fractures. Long-term prospective studies, real-world registries, and post-marketing surveillance are therefore needed to clarify the relationships among incretin therapies, bone remodeling, bone quality, muscle preservation, and fracture risk. Conclusively, to support clinical implementation, however, this multidimensional approach must be evaluated alongside clinical, pharmacological, and economic data, ensuring accurate risk stratification, feasibility, and rational allocation of healthcare resources.

Within this framework, urinary DPD represents a promising non-invasive adjunct for longitudinal assessment of bone resorption. However, current evidence is insufficient to support its routine clinical use as a standalone monitoring biomarker, given the influence of renal function, hydration status, biological variability, and the absence of validated fracture-predictive thresholds. Before widespread implementation, prospective studies should determine whether longitudinal DPD trajectories provide independent prognostic information beyond established serum biomarkers and whether they improve prediction of clinically meaningful skeletal outcomes in patients receiving incretin-based therapies. Most importantly, these considerations should not detract from the substantial metabolic, cardiovascular, renal, and weight-loss benefits of GLP-1 receptor agonists, which continue to outweigh potential skeletal concerns in appropriately selected patients.

## Figures and Tables

**Table 1:** Comparative Overview of Most Valuable Radiological and Biochemical Modalities for Multidimensional Osteoporosis Monitoring in Obesity, Type 2 Diabetes Mellitus, and Incretin-Based Therapies *.

Monitoring Type Method/Biomarker		Primary Molecular/Structural Mechanism Evaluated	Main Clinical Value	Prognostic Significance	Advantages	Major Limitations	Relevance in Obesity/T2DM/Incretin Therapies
**DXA**	Radiological	Quantitative BMD assessment reflecting cumulative mineralized bone mass	Gold-standard diagnosis and longitudinal monitoring	Strong predictor of fragility fracture risk	Widely available, reproducible, low radiation, standardized	Incompletely captures bone quality; affected by obesity, vascular calcifications, positioning errors, and device variability	May underestimate skeletal fragility in obesity and T2DM, where BMD is preserved despite impaired bone quality; limited sensitivity to rapid incretin-related remodeling
**TBS**	Radiological/computational	Trabecular microarchitectural texture derived from DXA gray-level analysis	Evaluates bone quality beyond BMD	Predicts fracture risk independent of BMD	Adds microarchitectural information without extra radiation	Influenced by obesity and soft tissue artifacts; indirect measure	Particularly valuable in T2DM and obesity, where trabecular deterioration occurs despite normal BMD
**VFA**	Radiological	Detection of occult vertebral compression fractures	Identifies silent vertebral fractures	Strong predictor of future fractures and mortality	Low radiation adjunct to DXA	Lower resolution than standard radiographs	Important in glucocorticoid users and high-risk metabolic populations
**HSA**	Radiological/computational	Geometric and biomechanical assessment of femoral strength and cortical distribution	Estimates structural resistance to loading	Predicts hip fracture susceptibility	Evaluates biomechanical competence beyond BMD	Limited routine clinical implementation	Useful in rapid weight-loss states with reduced skeletal loading
**HR-pQCT**	Advanced radiological	High-resolution cortical and trabecular microarchitecture; volumetric density	Research-level evaluation of bone quality and microstructural deterioration	Highly sensitive for early skeletal fragility	Distinguishes cortical vs. trabecular abnormalities; evaluates porosity	Expensive, limited accessibility, higher complexity	Particularly informative in T2DM, obesity, and incretin-induced skeletal remodeling
**P1NP**	Biochemical marker	Type I collagen synthesis and osteoblastic activity	Preferred marker for longitudinal monitoring and anabolic response	Early predictor of therapeutic efficacy and adherence	Low biological variability, minimal circadian fluctuation, relatively stable in renal dysfunction, standardized assays	Less sensitive to acute resorption changes	Valuable for monitoring anabolic adaptation during GLP-1RA-associated weight loss
**β** **-CTX**	Biochemical (resorption marker)	Osteoclastic collagen degradation and bone resorption dynamics	Preferred marker for antiresorptive response and rapid remodeling changes	Predicts accelerated bone loss and fracture-associated remodeling	Highly sensitive to therapeutic modulation	Significant circadian and feeding variability; fasting morning sampling required; influenced by renal dysfunction	Useful for detecting rapid catabolic remodeling induced by aggressive weight loss or dual incretin agonism
**Urinary DPD**	Biochemical (urinary resorption marker)	Cross-linked collagen degradation reflecting osteoclastic activity and bone resorption dynamics	Dynamic non-invasive monitoring of skeletal turnover and potential early detection of accelerated bone loss	May identify longitudinal changes in bone resorption, but its independent ability to predict future fractures has not yet been validated in patients receiving GLP-1RAs.	Non-invasive, patient-friendly, suitable for frequent outpatient assessment, potentially self-monitoring	Renal dependence, urinary dilution effects, circadian variability, less robust assay standardization, moderate intra-individual biological variability (~20–30%), limited assay harmonization compared with serum CTX/P1NP, absence of validated clinical cut-off values for fracture prediction or treatment guidance, reduced interpretability in elderly individuals, T2DM, and CKD populations	Particularly attractive for longitudinal monitoring during rapid weight loss or incretin-based therapy. Future development of rapid urine immunochromatographic assays—low-cost real-time self-monitoring of bone resorption

* Combining advanced imaging techniques with dynamic bone turnover biomarkers may improve early detection of remodeling imbalance, therapeutic monitoring, and individualized fracture-risk stratification. Importantly, the future development of rapid urinary DPD immunochemical self-tests could represent a transformative innovation in osteoporosis care. Abbreviations: BMD, bone mineral density; CKD, chronic kidney disease; CTX, C-terminal telopeptide of type I collagen; DPD, deoxypyridinoline; DXA, dual-energy X-ray absorptiometry; GLP-1RA, glucagon-like peptide-1 receptor agonist; HSA, hip structural analysis; HR-pQCT, high-resolution peripheral quantitative computed tomography; P1NP, procollagen type I N-terminal propeptide; T2DM, type 2 diabetes mellitus; TBS, trabecular bone score; VFA, vertebral fracture assessment.

## Data Availability

Not applicable.
